# Isolated diastolic hypertension and target organ damage: Findings from the STANISLAS cohort

**DOI:** 10.1002/clc.23713

**Published:** 2021-09-15

**Authors:** Luca Monzo, João Pedro Ferreira, Zohra Lamiral, Erwan Bozec, Jean‐Marc Boivin, Olivier Huttin, Marilucy Lopez‐Sublet, Nicolas Girerd, Faiez Zannad, Patrick Rossignol

**Affiliations:** ^1^ Université de Lorraine, Inserm, Centre d'Investigations Cliniques, ‐ Plurithématique 14‐33, and Inserm U1116, CHRU Nancy, F‐CRIN INI‐CRCT (Cardiovascular and Renal Clinical Trialists) Nancy France; ^2^ Department of Clinical, Internal, Anesthesiologic and Cardiovascular Sciences Sapienza University Rome Italy; ^3^ Department of Internal Medicine ESH Hypertension Excellence Centre, CHU Avicenne, AP‐HP, F‐CRIN INI‐CRCT Nancy France

**Keywords:** ambulatory blood pressure, isolated diastolic hypertension, target organ damage

## Abstract

**Background:**

Isolated diastolic hypertension (IDH) is defined as diastolic blood pressure (DBP) ≥80 mmHg and systolic blood pressure (SBP) <130 mmHg according to 2017 ACC/AHA guidelines. The effective cardiovascular risk linked to IDH is debated.

**Hypothesis:**

IDH might contribute marginally to hypertension‐related target organ damage (TOD) development.

**Methods:**

In this cross‐sectional analysis 1605 subjects from the STANISLAS cohort, a large familiar longitudinal study from Eastern France, were included. Participants were categorized according to average values at 24‐h ABP recording as having normal BP (SBP < 130/DBP < 80 mmHg); combined hypertension (SBP ≥130/DBP ≥80 mmHg or on antihypertensive treatment); IDH (SBP <130/DBP >80 mmHg); isolated systolic hypertension (ISH: SBP ≥130/DBP <80 mmHg). The association between hypertension status and TOD was assessed by multivariable‐adjusted logistic models.

**Results:**

Using normotension as reference, IDH was not significantly associated with NTproBNP levels (adjusted odds ratio [OR] 1.04 [95%CI 0.82;1.32], *p =* .750), microalbuminuria (OR 0.99 [0.69; 1.42], *p =* .960), diastolic dysfunction (OR 1.53 [0.88; 2.68], *p =* .130), left ventricular (LV) mass index (OR per 10 g/m^2^ increase 1.07 [0.95; 1.21], *p =* .250), LV longitudinal strain (global: OR 1.07 [0.99; 1.14], *p =* .054; subendocardial: OR 1.06 [0.99; 1.13], *p =* .087), carotid intima media thickness (OR 1.27 [0.79; 2.06], *p =* .320), reduced ankle‐brachial index (<0.9; OR 1.59 [0.19; 13.55], *p =* .670) and pulse wave velocity (PWV; OR 1.07 [0.93; 1.23], *p =* .360). In contrast, combined hypertension and ISH were independently associated with LV mass index and PWV increase (all *p ≤* .01).

**Conclusions:**

IDH was not significantly associated with TOD. Further studies are needed to clarify the clinical role of IDH. Registration: URL: https://www.clinicaltrials.gov; Unique identifier: NCT01391442.

## 1 INTRODUCTION

Hypertension is a major risk factor for cardiovascular morbidity and mortality.^1^ Current guidelines classified hypertension into isolated diastolic (IDH), isolated systolic (ISH), and systolic and diastolic mixed (or combined) hypertension based on the elevation of systolic and/or diastolic blood pressure (DBP) values.^2^, ^3^ IDH is a less prevalent hypertension definition,^4^ and is classified as elevated diastolic BP with a systolic BP within the normal range.^2^, ^3^ Thresholds for hypertension diagnosis are defined as office systolic BP values (SBP) ≥140 mmHg and/or diastolic BP values (DBP) ≥ 90 mmHg according to the 2018 European Society of Cardiology (ESC)/European Society of Hypertension (ESH) Guidelines.^3^ In 2017 American College of Cardiology (ACC)/American Heart Association (AHA) guidelines lowered the cut‐off from 140/90 to 130/80 mmHg.^2^ This change had major clinical and socioeconomic implications due to the increased number of eligible patients for treatment in a cohort classically defined at low risk for cardiovascular events.^5^, ^6^, ^7^ A recent longitudinal analysis that included 8703 adults failed to show a significant association between IDH, as defined by the 2017 ACC/AHA guidelines, and increased risk for cardiovascular outcomes.^8^ In line, previous observations demonstrated that IDH is usually not associated with cardiovascular outcomes independently of baseline systolic BP.^5^, ^9^, ^10^ On the other side, some studies showed a slight but significant association between diastolic hypertension and cardiovascular risk,^11^, ^12^, ^13^, ^14^ although the larger part of these reports did not exclusively investigate IDH but diastolic hypertension in the setting of combined hypertension. Given this context, the effective cardiovascular risk linked to IDH is debatable. The Suivi Temporaire Annuel Non‐Invasif de la Santé des Lorrains Assurés Sociaux (STANISLAS) cohort is a longitudinal transgenerational study from the Nancy region of France characterized by a familial structure and a long follow‐up (up to 23 years). In this cohort, individuals underwent to an extensive cardiovascular evaluation and the hypertensive status was evaluated by ambulatory BP monitoring (ABPM), that provides a more accurate diagnosis and a better prediction of cardiovascular risk compared to office BP.^2^, ^3^ Moreover, several papers have suggested that 24‐h average blood pressure (BP) is superior to office BP in relation to hypertension target organ damage.^15^ The STANISLAS cohort therefore offers the unique opportunity of studying the early changes induced by cardiovascular risk factors in initially healthy subjects.^16^ The aim of the present study is to determine the association between IDH, identified by 24‐h ambulatory BP monitoring, and markers of target organ damage in a populational cohort with detailed cardiovascular phenotyping and long follow‐up.

## 2 METHODS

### 2.1 Study population

This cross‐sectional study is derived from the STANISLAS cohort, a single‐center familial longitudinal cohort comprised of 1006 families (4295 subjects) from the Nancy region recruited in 1993–1995 at the Center for Preventive Medicine. The cohort was established with the primary objective of investigating gene–gene and gene–environment interactions in the field of cardiovascular diseases. The families were deemed healthy, free of declared acute and/or chronic illness, in order to assess the effect of genetics on the variability of intermediate phenotypes on the transition toward disease. From 2011 to 2019, 1705 survivors of the original cohort underwent their fourth examination (STANISLAS‐V4) at our department, as previously described.^17^ The research protocol was approved by the local Ethics Committee (Comité de Protection des Personnes Est III‐Nancy‐France) and all study participants gave a written informed consent to participate. The informed written consent was approved previously by the local ethics committee (ClinicalTrials.gov identifier NCT01391442).^16^

**FIGURE 1:**
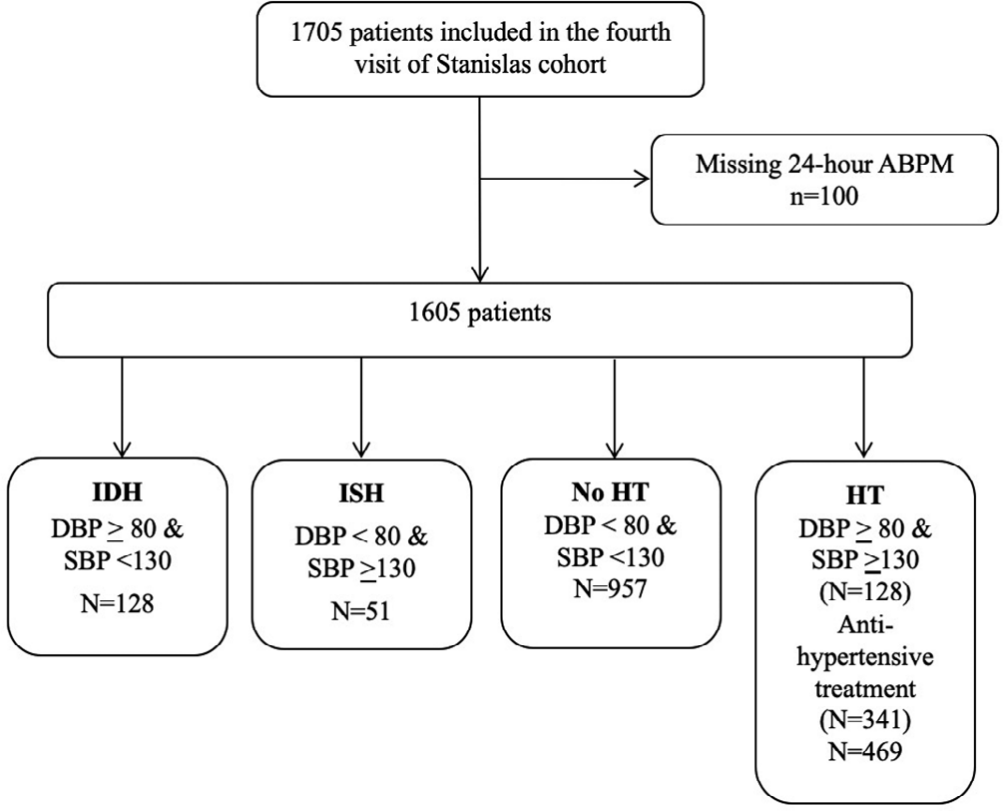
Study flowchart. ABPM, ambulatory blood pressure monitoring; DBP, diastolic blood pressure; HT, systolic‐diastolic hypertension; IDH, isolated diastolic hypertension; ISH, isolated systolic hypertension; SBP, systolic blood pressure

For the present study, 1605 adult patients (i.e., ≥18 years old and with ambulatory BP measurements) attending STANISLAS‐V4 were included in the analyses (Figure 1). All participants were scheduled to attend the Centre d'Investigation Clinique Plurithématique Pierre Drouin at Nancy University Hospital at 8 am after a 12‐ to 14‐h fast. All subjects underwent blood and urine sampling for laboratory analysis. Medical history, medications, anthropometric parameters were also recorded.

### 2.2 Blood pressure measurements

Office BP was measured three times in all participants, at 1‐min intervals, using an electronic sphygmomanometer after the participant had rested for at least 10 min and calculated as the mean of the three measurements. As previously described,^16^ all participants underwent a 24‐h recording of ABPM using the Spacelabs 90207 ambulatory monitor (Spacelabs Medical, WA). The monitoring cuff was placed around the participant's nondominant arm. The BP system was programmed to acquire measurements every 15 min from 6 am to 10 pm and every 30 min from 10 pm to 6 am. Self‐reported sleep–wake times were used to divide ABP monitoring data into daytime and nocturnal periods. BP indices were calculated from 24‐h, daytime, and nighttime averaged measurements according to current guidelines.^18^ In addition, participants had to complete a diary describing their main daily activities (e.g., eating, sleeping) and were asked to avoid excessive exercise during the 24‐h recording. Central reading of the recordings was performed by a trained technician blinded to the participants' clinical features. Data were considered for further analysis if they met the following criteria: the recording lasted ≥24 h, ≥70% of the expected number of readings were available, no missing data for >2 consecutive hourly intervals, and ≥2 valid measurements were obtained per hour. Mean 24 h diastolic and systolic BP were computed as the sum of non‐missing available BP values on the period of 24 h divided by the number of non‐missing readings recorded during the same period. Similarly, mean daytime and night‐time diastolic and systolic BP were calculated as the sum of non‐missing BP values recorded during day time or night‐time divided on the number of non‐missing readings recorded during the same period. Participants were categorized based on the average values at 24‐h ABP recording as having normal BP (SBP <130/DBP <80 mmHg; n = 957); combined hypertension (SDH; SBP ≥130 /DBP ≥80 mmHg or on antihypertensive treatment; n = 469); isolated diastolic hypertension (SBP <130/DBP ≥80 mmHg; n = 128); isolated systolic hypertension (SBP ≥130/DBP <80 mmHg; n = 51). The patient was considered a dipper if SBP and DBP reduced more than 10% between daytime and nighttime, otherwise he was considered a nondipper.^18^

### 2.3 Assessment of markers of target organ damage

Echocardiographic exams were performed by an experienced sonographer, in the left lateral decubitus position with a commercially available standard ultrasound scanner (Vivid 9, General Electric Medical Systems, Horten, Norway) using a 2.5 MHz phased‐array transducer (M5S), as previously described.^16^ The echo/Doppler examination was performed according to EAE/ASE recommendations^19^ and longitudinal left ventricular (LV) deformation parameters (strain) were obtained using speckle tracking echocardiography. LV diastolic dysfunction (DD) was assessed according to the 2009 ASE/EACVI recommendations.^20^ We used this algorithm instead of the most recent 2016 ASE/EACVI scoring system because the new criteria commonly results in a marked reduction of DD, as also recently demonstrated by our group.^21^ Image acquisitions were shown to be highly reproducible.^17^

Carotid intima media thickness (cIMT) measurements were performed by high‐resolution echo‐tracking (Wall Track System; Pie Medical, Maastricht, The Netherlands) on the right common carotid artery at 1–2 cm below the carotid bifurcation and the retained value was obtained as the mean of four measurements.^22^ Both reproducibility and agreement (intra/interoperator/devices) were excellent.^16^

Carotid to femoral pulse wave velocity (PWV) measurement was performed with Complior® (Alam Medical, Vincennes, France) and Sphygmocor® CVMS (AtCor Medical, Sydney, NSW, Australia) devices according to the European Network for Noninvasive Investigation of Large Arteries recommendations.^23^ The protocol was previously discussed in detail.^16^

The ankle‐brachial index (ABI) was calculated as the systolic pressure at the ankle, divided by the systolic pressure at the arm. Systolic BP was measured with a continuous Doppler machine using a BP cuff. An ABI between 0.9 and 1.2 is considered normal, while an index <0.9 suggests the presence of arterial disease.^24^ The measurements were performed according to a standardized protocol^25^ on the side (arm and ankle) with the highest SBP.

N‐terminal pro–B‐type natriuretic peptide (NT‐proBNP) was measured using a multiplex assay (CVDII panel, Olink Proteomics AB®, Uppsala, Sweden). The final assay readout was expressed as normalized protein expression values, which is an arbitrary unit on a log2 scale in which a higher value corresponds to higher protein expression.

### 2.4 Statistical methods

Proportions were compared using 𝜒^2^ test and were expressed as number (proportion as percentage). Continuous variables were expressed as mean ± SD or median (interquartile range [IQR: Q1; Q3]) and compared using one‐way ANOVA and Kruskal–Wallis depending on the normality of the distribution. We focused on the association between noninvasive markers of target organ damage and IDH, with a cutoff of DBP ≥80 mmHg and SBP <130 mmHg at 24‐h ABP recording. Descriptive statistics were computed on all patients' characteristics in the sample overall and according to hypertension status. Multinomial logistic regression models were used to assess the associations between the dependent variable hypertension (HT) categories (No HT [reference], IDH, ISH, and SDH) and independent variables (echocardiographic, vascular and biological markers of target organ damage). The log linearity assumptions of the relationship between HT status and the continuous variables of target organ damage were assessed using restricted cubic splines according to the Harrell's rule.^26^ We use the Wald test for assessing the linearity assumptions. The variables were then categorized, as required, to meet the model assumptions. Each model was adjusted for gender, age, waist circumference, smoking status, total cholesterol, glycemia, lipid lowering agents, self‐declared hypertension status, glomerular filtration rate, and hemoglobin. The two‐tailed significance level was set at *p <* .05. All analyses were performed using SAS version 9.4.6 (SAS Institute Inc., Cary, NC) and R version 3.6.1 (2019‐07‐05).

## 3 RESULTS

### 3.1 Participants' baseline characteristics

The characteristics of the population are given in Table 1. The study examined 1605 subjects, 128 with IDH, 51 with ISH, 469 with SDH and 957 without hypertension. Overall, the mean age was 56 (IQR 35; 60) years, 48.2% were male, the mean BMI was 25.2 (IQR 22.5; 28.6) kg/m^2^ and the mean estimated glomerular filtration rate was 96.1 [86.7; 106.6] ml/min/1.73 m^2^. A history of diabetes, hypercholesterolemia and current smoking habit was identified in the 4.2%, 16%, and 20.7% of the sample, respectively. Participants with IDH were more likely to be younger, women and current smoker than those who had other types of hypertension, but not of normotensives. Non‐dipper BP pattern was slightly more prevalent in IDH and ISH compared with SDH.

**TABLE 1:** Population characteristics in the overall cohort and according to hypertension status

Characteristics	Overall (N = 1605)	IDH (N = 128)	ISH (N = 51)	SDH (N = 469)	No hypertension (N = 957)	*p*‐value
		SBP <130/DBP >80 mmHg	SBP ≥130/DBP <80 mmHg	SBP ≥130/DBP ≥80 mmHg	SBP <130/DBP <80 mmHg	
Age, years	56 [35; 60]	55 [36; 59]	58 [33; 63]	59 [56; 63]	41 [33; 59]	<.0001^a^
Male, n (%)	774 (48.2)	75 (58.6)	35 (68.6)	282 (60.1)	382 (39.9)	<.0001^a^
BMI, Kg/m^2^	25.2 [22.5; 28.6]	25.0 [22.3; 27.8]	24.9 [23.8; 28.3]	27.8 [24.8; 31.8]	24.2 [21.9; 27.3]	<.0001^a^
Waist circumference (cm)	90.0 [80.0; 99.0]	91.0 [81.0; 98.0]	91.0 [85.0; 99.0]	97.0 [89.0; 106.0]	85.0 [77.0; 94.0]	<.0001^a^
Diabetes, n (%)	67 (4.2)	2 (1.6)	0 (0.0)	48 (10.3)	17 (1.8)	<.0001^a^
Hypertension duration, years	NA	2.5 [2.0; 9.0]	5.0 [1.0; 10.0]	8.0 [4.0; 13.0]	NA	.13
PVD, n (%)	8 (0.5)	0 (0.0)	1 (2.0)	4 (0.9)	3 (0.3)	.19
Smoker status, n (%)						
Current Former Never	330 (20.7) 517 (32.4) 751 (47.0)	24 (18.8) 45 (35.2) 59 (46.1)	9 (18.0) 12 (24.0) 29 (58.0)	73 (15.7) 202 (43.4) 190 (40.9)	224 (23.5) 258 (27.0) 473 (49.5)	<.0001^a^
Heart rate, bpm	63 (58; 69)	64 (59; 70)	61 (54; 70)	64 (58; 70)	63 (58; 69)	.15
Hemoglobin, g/L	14.71 ± 1.20	15.04 ± 1.11	14.91 ± 1.12	14.86 ± 1.22	14.59 ± 1.19	<.0001^a^
Total cholesterol, g/L	2.1 ± 0.4	2.1 ± 0.3	2.0 ± 0.4	2.1 ± 0.4	2.1 ± 0.4	.3
LDL, g/L	1.34 ± 0.34	1.34 ± 0.31	1.29 ± 0.35	1.34 ± 0.36	1.34 ± 0.33	.75
HDL, g/L	0.57 [0.48; 0.66]	0.56 [0.47; 0.67]	0.54 [0.45; 0.61]	0.54 [0.45; 0.62]	0.58 [0.49; 0.68]	<.0001^a^
Triglycerides, g/L	0.92 [0.68; 1.27]	0.93 [0.65; 1.39]	0.87 [0.66; 1.14]	1.12 [0.81; 1.49]	0.85 [0.64; 1.14]	<.0001^a^
Glycemia, g/L	0.88 [0.83; 0.95]	0.88 [0.81; 0.93]	0.89 [0.85; 0.95]	0.94 [0.87; 1.03]	0.87 [0.81; 0.92]	<.0001^a^
eGFR CKD‐EPI, ml/min/1.73m^2^	96.1 [86.7; 106.6]	95.7 [87.7; 105.7]	95.0 [87.0; 107.7]	91.4 [79.7; 99.2]	98.2 [89.4; 110.4]	<.0001^a^
C‐reactive protein, mg/L	1.5 [0.7; 3.2]	1.5 [0.9; 3.3]	1.1 [0.7; 3.0]	2.0 [1.0; 3.7]	1.3 [0.7; 2.8]	<.0001^a^
Microalbuminuria, mg/L	6.0 [3.6; 9.9]	6.4 [3.4; 11.2]	6.1 [3.4; 11.9]	6.6 [3.8; 11.6]	5.7 [3.5; 9.1]	.009^a^
NT‐ProBNP (Olink)	3.55 [2.87; 4.28]	3.40 [2.81; 4.27]	3.66 [2.63; 4.36]	3.68 [3.04; 4.55]	3.49 [2.81; 4.17]	<.0001^a^
Lipid lowering therapy, n (%)	257 (16.0)	9 (7.0)	8 (15.7)	172 (36.7)	68 (7.1)	<.0001^a^
Diabetes treatment, n (%)	59 (3.7)	2 (1.6)	0 (0.0)	46 (9.8)	11 (1.1)	<.0001^a^
Antihypertensive drugs, n (%)	341 (21.2)	0	0	341 (72.7)	0	NA
^b^Non‐dipper status, n (%)	669 (48.6)	60 (57.1)	25 (61.0)	174 (44.7)	410 (48.7)	.048
LV‐GLS, %	−20.8 [−22.5; −18.8]	−19.9 [−22.0; −18.4]	−19.9 [−21.6; −18.1]	−20.3 [−22.2; −17.9]	−21.1 [−22.7; −19.2]	<.0001^a^
Left ventricular mass index, g/m^2^	73.9 [62.8; 87.4]	73.5 [64.4; 87.1]	83.6 [71.6; 94.9]	82.4 [71.9; 96.5]	69.1 [59.4; 81.6]	<.0001^a^
Left ventricular hypertrophy, n (%)	233 (14.7)	18 (14.4)	10 (20.4)	109 (23.7)	96 (10.1)	<.0001^a^
Left atrial volume index, ml/m^2^	21.9 [17.7; 26.5]	21.3 [17.5; 25.7]	22.5 [19.0; 26.6]	23.7 [18.6; 28.9]	21.3 [17.3; 25.6]	<.0001^a^
E/e' ratio	6.2 [5.2; 7.4]	6.2 [5.3; 7.5]	6.6 [5.9; 8.4]	7.1 [6.0; 8.4]	5.8 [5.0; 6.8]	<.0001^a^
E/A ratio	1.10 [0.87; 1.41]	1.07 [0.84; 1.28]	1.19 [0.93; 1.48]	0.91 [0.78; 1.13]	1.20 [0.96; 1.53]	<.0001^a^
Diastolic dysfunction, n (%)	293 (18.7)	23 (18.5)	10 (20.0)	157 (34.2)	103 (11.0)	<.0001^a^
LVEDV index, ml/m^2^	47.8 [40.0; 56.5]	48.9 [39.1; 61.4]	47.4 [39.2; 61.7]	47.1 [38.9; 57.2]	47.8 [40.4; 55.7]	.35
LVESV index, ml/m^2^	16.3 [12.9; 20.5]	17.4 [12.9; 22.1]	16.4 [13.3; 22.5]	15.8 [11.9; 20.2]	16.5 [13.4; 20.4]	.023^a^
Left ventricular ejection fraction, %	65.4 [61.2; 69.4]	65.4 [60.2; 69.8]	66.4 [60.1; 70.8]	66.4 [62.1; 70.8]	64.9 [61.0; 68.7]	.0009^a^
Pulse wave velocity, m/s	8.2 [7.3; 9.3]	8.4 [7.6; 9.1]	9.2 [7.8; 10.3]	9.1 [8.0; 10.3]	7.8 [7.1; 8.8]	<.0001^a^
Carotid intima media thickness, μm	616.0 [526.0; 717.2]	634.3 [537.0; 722.0]	628.0 [544.0; 766.0]	692.0 [603.2; 791.0]	579.0 [500.0; 674.0]	<.0001^a^
Ankle‐brachial index < 0.9, n (%)	16 (1.0)	1 (0.8)	1 (2.0)	5 (1.1)	9 (1.0)	.90

*Note*: Data are presented as mean ± SD or median and interquartile range [Q1; Q3].

Abbreviations: BMI, body mass index; CV, cardiovascular; DBP, diastolic blood pressure; eGFR, estimated glomerular filtration rate; GLS, global longitudinal strain; IDH, isolated diastolic hypertension; LV, left ventricular; LVEDV, left ventricular end‐diastolic volume; LVESV, left ventricular end‐systolic volume; NA, not applicable; PVD, peripheral vascular disease; SBP, systolic blood pressure; SDH, systolic‐diastolic mixed hypertension.

^a^ Statistically significant difference between groups.

^b^ 1377 patients available for analysis.

Subjects with IDH showed higher prevalence of left ventricular hypertrophy and diastolic dysfunction compared to normotensives, but lower than other groups of hypertensives. Among other markers of target organ damage, microalbuminuria level and cIMT were higher in participants with IDH compared to those without hypertension and ISH, but lower than SDH. On the contrary, estimated glomerular filtration rate and LV‐GLS showed lower values in IDH compared to normotensives, and intermediate between ISH and SDH. Finally, participants with IDH showed the lower value of NTproBNP and similar prevalence of pathologically reduced ABI compared to other BP categories.

### 3.2 Associations between hypertension classes and markers of target organ damage

**FIGURE 2:**
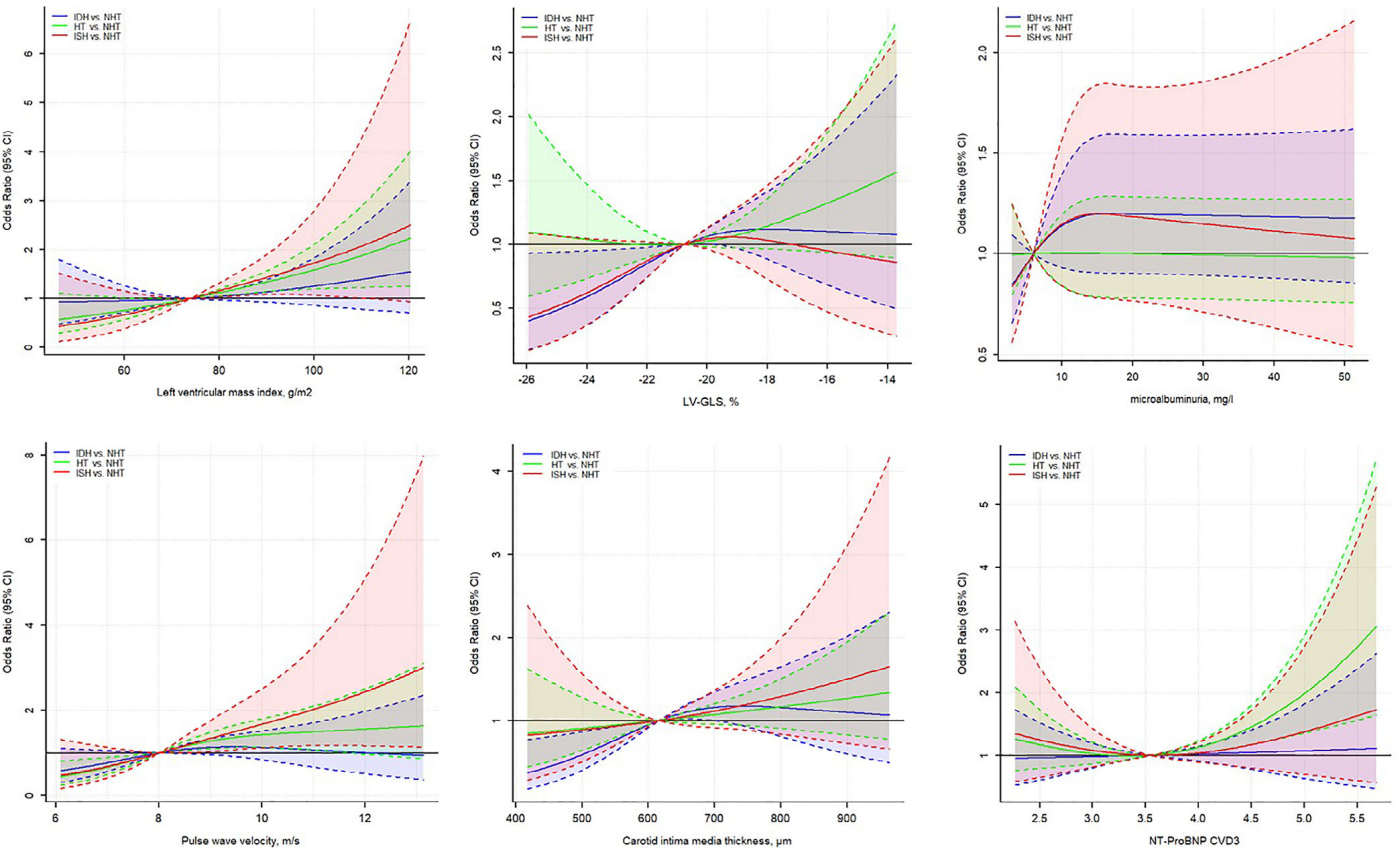
“Spline” graphical representation of the association between hypertension categories and markers of target organ damage. Solid line: hazard ratio; dashed lines: 95% confidence interval. Blue line: isolated diastolic hypertension (IDH); green line: systolic‐diastolic hypertension (HT); red line: isolated systolic hypertension (ISH). GLS, global longitudinal strain

In models adjusted for gender, age, waist circumference, smoking status, total cholesterol, glycemia, lipid lowering agents, self‐declared hypertension status, glomerular filtration rate and hemoglobin, the majority of associations in the “crude model” between IDH and markers of target organ damage became no longer significant (Table 2). In particular, with normotension as reference, IDH was not significantly associated with NTproBNP levels (*p =* .750), microalbuminuria (*p =* .960), diastolic dysfunction (*p =* .130), LV mass index (*p =* .250), LV longitudinal strain (global, *p =* .054; subendocardial, *p =* .087), cIMT (*p =* .320), pathologically reduced ABI (<0.9; *p =* .670) and PWV (*p =* .360). In contrast, mixed hypertension and ISH appeared strongly and directly associated with LV mass index and PWV increase, meanwhile only SDH was associated with NTproBNP concentrations (all *p ≤* .010). Using spline‐based analyses, we did not find any evidence of a nonlinear association between hypertension classes (compared to normal BP) and markers of target organ damage, except for the association between SDH and NTproBNP (Figure 2). Sensitivity analysis based on office BP measurements showed similar findings (Table S1).

**TABLE 2:** Crude and adjusted association between markers of target organ damage and hypertension categories

	Blood pressure categories	Univariate		Multivariable^c^	
		Odds ratio [95% CI]	*p*‐value	Odds ratio [95% CI]	*p*‐value
	Reference: no hypertension	1.00		1.00	
Left ventricular mass index, per increment of 10 g/m^2^	Systolic‐diastolic hypertension	1.49 [1.40; 1.59]	<.0001^a^	1.19 [1.09; 1.32]	.0003^a^
	Isolated diastolic hypertension	1.19 [1.07; 1.32]	.001^a^	1.07 [0.95; 1.21]	.250
	Isolated systolic hypertension	1.41 [1.22; 1.63]	<.0001^a^	1.25 [1.05; 1.47]	.010^a^
Left ventricular hypertrophy, n (%)	Systolic‐diastolic hypertension	2.76 [2.05; 3.74]	<.0001^a^	1.52 [0.98; 2.35]	.064
	Isolated diastolic hypertension	1.49 [0.87; 2.57]	.150	1.22 [0.69; 2.18]	.490
	Isolated systolic hypertension	2.28 [1.10; 4.70]	.026^a^	2.03 [0.4; 4.39]	.073
Left atrial volume index, per increment of 10 ml/m^2^	Systolic‐diastolic hypertension	1.67 [1.43; 1.95]	<.0001^a^	1.39 [1.11; 1.77]	.005^a^
	Isolated diastolic hypertension	0.98 [0.74; 1.30]	.910	0.86 [0.64; 1.17]	.340
	Isolated systolic hypertension	1.45 [0.99; 2.11]	.054	1.22 [0.81; 1.84]	.340
LV‐GLS, %	Systolic‐diastolic hypertension	1.11 [1.07; 1.16]	<.0001^a^	1.04 [0.98; 1.10]	.170
	Isolated diastolic hypertension	1.09 [1.03; 1.16]	.006^a^	1.07 [0.99; 1.14]	.054
	Isolated systolic hypertension	1.09 [0.99; 1.19]	.057	1.04 [0.94; 1.15]	.450
Subendocardial LV‐GLS, %	Systolic‐diastolic hypertension	1.09 [1.06; 1.14]	<.0001^a^	1.03 [0.97; 1.09]	.270
	Isolated diastolic hypertension	1.08 [0.99; 1.17]	.012^a^	1.06 [0.99; 1.13]	.087
	Isolated systolic hypertension	1.08 [0.99; 1.17]	.075	1.03 [0.94; 1.13]	.510
E/e' ratio	Systolic‐diastolic hypertension	1.56 [1.45; 1.68]	<.0001^a^	1.17 [1.05; 1.29]	.003^a^
	Isolated diastolic hypertension	1.19 [1.06; 1.34]	.003^a^	1.15 [1.01; 1.30]	.034^a^
	Isolated systolic hypertension	1.42 [1.23; 1.65]	<.0001^a^	1.36 [1.15; 1.60]	.0002^a^
E/A ratio	Systolic‐diastolic hypertension	0.13 [0.09; 0.18]	<.0001^a^	0.85 [0.48; 1.50]	.570
	Isolated diastolic hypertension	0.47 [0.29; 0.77]	.003^a^	0.69 [0.36; 1.31]	.250
	Isolated systolic hypertension	0.87 [0.45; 1.69]	.69	1.65 [0.79; 3.46]	.180
Diastolic dysfunction, n (%)	Systolic‐diastolic hypertension	4.19 [3.17; 5.56]	<.0001^a^	1.53 [0.99; 2.35]	.055
	Isolated diastolic hypertension	1.84 [1.12; 3.02]	.016^a^	1.53 [0.88; 2.68]	.130
	Isolated systolic hypertension	2.02 [0.98; 4.16]	.057	1.60 [0.71; 3.62]	.260
LVEDV index, per increment of 10 ml/m^2^	Systolic‐diastolic hypertension	0.99 [0.91; 1.08]	.850	0.99 [0.87; 1.14]	.980
	Isolated diastolic hypertension	1.13 [0.98; 1.29]	.096	1.13 [0.96; 1.32]	.140
	Isolated systolic hypertension	1.11 [0.90; 1.37]	.320	0.99 [0.79; 1.25]	.970
LVESV index, per increment of 10 ml/m^2^	Systolic‐diastolic hypertension	0.90 [0.75; 1.08]	.260	1.14 [0.88; 1.47]	.320
	Isolated diastolic hypertension	1.23 [0.95; 1.59]	.120	1.23 [0.92; 1.65]	.170
	Isolated systolic hypertension	1.13 [0.75; 1.70]	.560	0.98 [0.62; 1.55]	.930
^b^LV ejection fraction, %	Systolic‐diastolic hypertension	1.55 [1.23; 1.94]	.0002^a^	0.99 [0.70; 1.39]	.950
	Isolated diastolic hypertension	1.22 [0.83; 1.77]	.310	1.14 [0.76; 1.69]	.530
	Isolated systolic hypertension	1.39 [0.78; 2.44]	.260	1.30 [0.71; 2.37]	.390
^b^Carotid intima media thickness, μm	Systolic‐diastolic hypertension	3.94 [3.07; 5.05]	<.0001^a^	1.05 [0.71; 1.57]	.800
	Isolated diastolic hypertension	1.61 [1.11; 2.34]	.012^a^	1.27 [0.79; 2.06]	.320
	Isolated systolic hypertension	1.55 [0.88; 2.73]	.130	0.85 [0.42; 1.76]	.670
Pulse wave velocity, m/s	Systolic‐diastolic hypertension	1.57 [1.46; 1.69]	<.0001^a^	1.17 [1.05; 1.31]	.006^a^
	Isolated diastolic hypertension	1.21 [1.08; 1.37]	.002^a^	1.07 [0.93; 1.23]	.360
	Isolated systolic hypertension	1.49 [1.29; 1.74]	<.0001^a^	1.30 [1.08; 1.56]	.005^a^
Ankle‐brachial index < 0.9	Systolic‐diastolic hypertension	1.14 [0.38; 3.42]	.81	2.03 [0.36; 11.40]	.42
	Isolated diastolic hypertension	0.85 [0.11; 6.73]	.87	1.59 [0.19; 13.55]	.67
	Isolated systolic hypertension	2.12 [0.26; 17.09]	.48	4.49 [0.49; 41.44]	.19
Microalbuminuria, per increment of 100 mg/L	Systolic‐diastolic hypertension	1.25 [0.97; 1.61]	.089	0.94 [0.74; 1.21]	.630
	Isolated diastolic hypertension	0.99 [0.54; 1.85]	1.00	0.99 [0.69; 1.42]	.960
	Isolated systolic hypertension	0.97 [0.34; 2.75]	.950	0.87 [0.36; 2.08]	.750
C‐reactive protein, per increment of 10 mg/L	Systolic‐diastolic hypertension	1.54 [1.23; 1.92]	.0002^a^	1.25 [0.92; 1.71]	.160
	Isolated diastolic hypertension	1.28 [0.89; 1.83]	.170	1.32 [0.93; 1.87]	.120
	Isolated systolic hypertension	0.91 [0.39; 2.08]	.820	0.91 [0.41; 2.02]	.810
NT‐ProBNP, (Olink)	Systolic‐diastolic hypertension	1.40 [1.246; 1.58]	<.0001^a^	1.29 [1.07; 1.56]	.009^a^
	Isolated diastolic hypertension	0.99 [0.81; 1.13]	.950	1.04 [0.82; 1.32]	.750
	Isolated systolic hypertension	1.03 [0.76; 1.39]	.830	1.04 [0.74; 1.47]	.820

Abbreviations: GLS, global longitudinal strain; LV, left ventricular; LVEDV, left ventricular end‐diastolic volume; LVESV, left ventricular end‐systolic volume.

^a^ Statistically significant.

^b^ Log linearity assumption not respected. Variable categorized according to the median value (≥median).

^c^ Multivariate adjusted for: gender, age, waist circumference, smoking status, total cholesterol, glycemia, lipid lowering agents, hypertension status, glomerular filtration rate, and hemoglobin.

## 4 DISCUSSION

The key finding of our study was that an isolated increase in diastolic BP, without a concurrent rise in systolic values at 24‐h ABP recording, was not significantly associated with markers of target organ damage. Concurrently, we showed that combined hypertension was associated with a higher NTproBNP concentration, meanwhile both SDH and ISH were correlated with LV mass index and PWV increase.

While a raise in systolic BP was previously reported to be associated with an increased risk of cardiovascular disease and adverse outcomes,^27^ diastolic hypertension seems to generally confer a low cardiovascular risk.^5^, ^6^, ^7^ In a recent longitudinal analysis that included 8703 US adults from National Health and Nutrition Examination Survey (NHANES) III (1988–1994), NHANES 1999–2014 and the Give Us a Clue to Cancer and Heart Disease (CLUE) II cohort, there was no significant association between IDH, as defined by the 2017 ACC/AHA guidelines (diastolic BP ≥ 80 mmHg), and incident atherosclerotic cardiovascular disease, heart failure, or chronic kidney disease.^8^ Another study conducted by ABP monitoring on 8.341 untreated subjects showed that IDH (diastolic BP ≥80 mmHg) did not increase the risk of total mortality, cardiovascular mortality or stroke, while ISH (systolic BP ≥130 mmHg) and mixed hypertension were associated with increased cardiovascular risk.^28^ It should be noted that few other studies showed an association between diastolic hypertension and cardiovascular risk,^11^, ^12^, ^13^, ^14^ together with a beneficial effect of antihypertensive treatment on cardiovascular mortality and morbidity. Although these evidences might appear in contradiction with the abovementioned observations, the larger part of these reports did not exclusively investigate IDH but diastolic hypertension in the setting of SDH, without differentiating the effect of solely diastolic or systolic BP lowering.

Subtle damage to certain organs can be detected in hypertensive patients early in the disease and can occur years before overt clinical events occur.^29^ Evaluation of markers of target organ damage constitute an easy to assess, early and reliable surrogate of hypertension‐related clinical events.^24^ In fact, it has been shown that target organ damage have an independent prognostic significance and strongly increases cardiovascular risk, irrespectively of the involved structure (heart, kidney, brain, or vessels).^3^ Wei et al.^30^ explored the association of target organ damage with 24‐h systolic and diastolic BP levels and ambulatory hypertension subtypes in a large cohort of untreated Chinese patients. They found that 24‐h SBP and mixed hypertension were the major determinants of target organ damage and its severity irrespective of age and target organ, whereas 24‐h diastolic BP and IDH only related to the urinary albumin/creatinine ratio below middle age. Another study by Lin et al.^31^ investigated the impact of ambulatory ISH, SDH and IDH (cut‐off for systolic BP ≥ 140 mmHg and diastolic BP ≥ 90 mmHg) on target organ damage in a population of 171 subjects, showing that IDH had only a marginal effect in determining target organ damage when compared to ISH and mixed hypertension. In a large metanalysis that involved 2485 patients with and without hypertension, SBP was more closely associated with target organ damage (LV mass index, cIMT, PWV, and urinary protein excretion) compared to diastolic BP.^32^ In a study conducted in 972 hypertensive patients at high cardiovascular risk, systolic BP was showed to be linearly associated with ABI, while diastolic BP was not.^33^ In line, Powell et al.^34^ showed a strong association between ISH and SDH and incident symptomatic peripheral artery disease in middle‐aged and older American women, whereas IDH was not associated with an increased risk. Finally, in a previous analysis of STANISLAS cohort, Ferreira et al.^22^ showed a linear positive association between systolic BP increase and cIMT thickness, meanwhile the association with diastolic BP was weaker and not significant.

We also demonstrated that, with normotension as reference, combined hypertension was associated with a higher NTproBNP concentration, meanwhile both SDH and ISH, but not IDH, were correlated with LV mass index and PWV increase. Prior observations suggested that LV mass is more closely related to systolic BP, whereas LV wall thickness correlates better with diastolic BP.^32^, ^35^, ^36^ Our finding is in line with previous evidences and corroborates the hypothesis that wall stress is mainly related to systolic BP and is a key determinant of LV hypertrophy development.^37^ Concurrently, LV wall stress was demonstrated to be a main determinant of natriuretic peptides increase,^38^ likely explaining the described correlation between mixed hypertension and NTproBNP. Although natriuretic peptides are not classically regarded as a marker of target organ damage, recent evidences showed that plasma NT‐proBNP is a strong prognostic marker in hypertensive patients.^39^ Systolic BP and arterial stiffness have been demonstrated to be closely related and may behave reciprocally as cause or effect, interacting in a vicious cycle. In this regard, PWV is considered a reliable measure to quantify arterial stiffness.^40^ Our results confirm previous observations showing the association between systolic BP, but not diastolic BP, and PWV.^41^

In our analysis we found a trend toward the association between GLS change and IDH. GLS has been demonstrated as a sensitive tool to recognize early subclinical systolic dysfunction in newly diagnosed hypertensive patients without LVH,^42^ even when ejection fraction and other strain components are normal.^43^ Interestingly, a recent echocardiographic study conducted on STANISLAS cohort identified a significant association between layer‐specific strain variables and self‐reported dyspnea, suggesting that refined strain components (i.e., subendocardial strain) could help identify early stages of increased LV filling pressure.^44^

Altogether, our results appear to be consistent with previous observations showing a lack of association between IDH and target organ damage. These findings might also provide an explanation for the inconclusive association between IDH and adverse cardiovascular outcomes frequently reported in the scientific literature.

### 4.1 Limitations

The main limitation of our study is its observational design; therefore, it is not possible to establish a causal link to the results obtained. In addition, given our sample size, we could not adjust our analysis for every possible cardiovascular risk variable. The cross‐sectional design allowed us to assess only intermediate signs of target organ damage; therefore, our findings cannot be extrapolated to the incidence of hard cardiovascular or renal end‐points. We had no assessment of microvascular damage besides microalbuminuria and therefore cannot rule out the impact of IDH on this target. The reclassification of our population according to ESC/ESH BP thresholds^3^ identified a very small number of IDH patients (N = 11), preventing any reliable sensitivity analysis in this setting (Figure S1). Different duration of hypertension among subgroups might underestimated the effect of BP on TOD in patients with a shorter follow‐up, although it was not statistically significant. Finally, the results of this analysis cannot be extended to general hypertensive population, as they refer to an initially healthy population with a low cardiovascular risk at the time of evaluation and constituted by a mix of already diagnosed and newly‐diagnosed hypertensives during STANISLAS research visit.

### 4.2 Conclusions

In our study IDH was not significantly associated with target organ damage. This finding might suggest that the clinical significance of IDH in the absence of elevated systolic BP is questionable. Further studies are needed to clarify the causative role of IDH in the development of target organ damage and cardiovascular outcomes.

## CONFLICT OF INTEREST

The authors declare no conflicts of interest.

## Supporting information


**Figure S1**: Patients allocation according to hypertension categories defined by ESC/ESH criteria. Blue, no hypertension (no HT); orange, systolic‐diastolic hypertension (SDH); gray, isolated diastolic hypertension (IDH); yellow, isolated systolic hypertension (ISH).

**Table S1**: Crude and adjusted association between markers of target organ damage and hypertension categories (office BP measurement).

## Data Availability

The data that support the findings of this study are available from the corresponding author upon reasonable request. All data will be made available upon request.

## References

[1] Lawes CM , Vander Hoorn S , Rodgers A . International society of H. global burden of blood‐pressure‐related disease, 2001. Lancet. 2008;371(9623):1513‐1518. 18456100 10.1016/S0140-6736(08)60655-8

[2] Whelton PK , Carey RM , Aronow WS , et al. 2017 ACC/AHA/ AAPA/ABC/ACPM/AGS/APhA/ASH/ASPC/NMA/PCNA guideline for the prevention, detection, evaluation, and management of high blood pressure in adults: executive summary: a report of the American College of Cardiology/American Heart Association task force on clinical practice guidelines. J Am Coll Cardiol. 2018;71(19):2199‐2269. 29146535 10.1016/j.jacc.2017.11.006

[3] Williams B , Mancia G , Spiering W , et al. 2018 ESC/ESH guidelines for the management of arterial hypertension. Eur Heart J. 2018;39(33):3021‐3104. 30165516 10.1093/eurheartj/ehy339

[4] Franklin SS , Jacobs MJ , Wong ND , L'Italien GJ , Lapuerta P . Predominance of isolated systolic hypertension among middle‐aged and elderly US hypertensives: analysis based on National Health and nutrition examination survey (NHANES) III. Hypertension. 2001;37(3):869‐874. 11244010 10.1161/01.hyp.37.3.869

[5] Fang J , Madhavan S , Cohen H , Alderman MH . Isolated diastolic hypertension. A favorable finding among young and middle‐aged hypertensive subjects. Hypertension. 1995;26(3):377‐382. 7649569 10.1161/01.hyp.26.3.377

[6] Petrovitch H , Curb JD , Bloom‐Marcus E . Isolated systolic hypertension and risk of stroke in Japanese‐American men. Stroke. 1995;26(1):25‐29. 7839392 10.1161/01.str.26.1.25

[7] Strandberg TE , Salomaa VV , Vanhanen HT , Pitkala K , Miettinen TA . Isolated diastolic hypertension, pulse pressure, and mean arterial pressure as predictors of mortality during a follow‐up of up to 32 years. J Hypertens. 2002;20(3):399‐404. 11875306 10.1097/00004872-200203000-00014

[8] McEvoy JW , Daya N , Rahman F , et al. Association of isolated diastolic hypertension as defined by the 2017 ACC/AHA blood pressure guideline with incident cardiovascular outcomes. JAMA. 2020;323(4):329‐338. 31990314 10.1001/jama.2019.21402 PMC6990938

[9] Hozawa A , Ohkubo T , Nagai K , et al. Prognosis of isolated systolic and isolated diastolic hypertension as assessed by self‐measurement of blood pressure at home: the Ohasama study. Arch Intern Med. 2000;160(21):3301‐3306. 11088093 10.1001/archinte.160.21.3301

[10] Nielsen WB , Lindenstrom E , Vestbo J , Jensen GB . Is diastolic hypertension an independent risk factor for stroke in the presence of normal systolic blood pressure in the middle‐aged and elderly? Am J Hypertens. 1997;10(6):634‐639. 9194509 10.1016/s0895-7061(96)00505-5

[11] Niiranen TJ , Rissanen H , Johansson JK , Jula AM . Overall cardiovascular prognosis of isolated systolic hypertension, isolated diastolic hypertension and pulse pressure defined with home measurements: the Finn‐home study. J Hypertens. 2014;32(3):518‐524. 24477096 10.1097/HJH.0000000000000070

[12] Sheriff HM , Tsimploulis A , Valentova M , et al. Isolated diastolic hypertension and incident heart failure in community‐dwelling older adults: insights from the cardiovascular health study. Int J Cardiol. 2017;238:140‐143. 28343761 10.1016/j.ijcard.2017.02.142 PMC6454920

[13] Lee H , Yano Y , Cho SMJ , et al. Cardiovascular risk of isolated systolic or diastolic hypertension in young adults. Circulation. 2020;141(22):1778‐1786. 32479205 10.1161/CIRCULATIONAHA.119.044838

[14] MacMahon SW , Cutler JA , Furberg CD , Payne GH . The effects of drug treatment for hypertension on morbidity and mortality from cardiovascular disease: a review of randomized controlled trials. Prog Cardiovasc Dis. 1986;29(3):99‐118. 3538183 10.1016/0033-0620(86)90038-1

[15] Mancia G , Parati G . Ambulatory blood pressure monitoring and organ damage. Hypertension. 2000;36(5):894‐900. 11082163 10.1161/01.hyp.36.5.894

[16] Ferreira JP , Girerd N , Bozec E , et al. Cohort profile: rationale and design of the fourth visit of the STANISLAS cohort: a familial longitudinal population‐based cohort from the Nancy region of France. Int J Epidemiol. 2018;47(2):395. 29220499 10.1093/ije/dyx240

[17] Frikha Z , Girerd N , Huttin O , et al. Reproducibility in echocardiographic assessment of diastolic function in a population based study (the STANISLAS cohort study). PLoS One. 2015;10(4):e0122336. 25853818 10.1371/journal.pone.0122336 PMC4390157

[18] O'Brien E , Parati G , Stergiou G , et al. European society of hypertension position paper on ambulatory blood pressure monitoring. J Hypertens. 2013;31(9):1731‐1768. 24029863 10.1097/HJH.0b013e328363e964

[19] Lang RM , Badano LP , Mor‐Avi V , et al. Recommendations for cardiac chamber quantification by echocardiography in adults: an update from the American Society of Echocardiography and the European Association of Cardiovascular Imaging. J Am Soc Echocardiogr. 2015;28(1):1‐39. 25559473 10.1016/j.echo.2014.10.003

[20] Nagueh SF , Appleton CP , Gillebert TC , et al. Recommendations for the evaluation of left ventricular diastolic function by echocardiography. Eur J Echocardiogr. 2009;10(2):165‐193. 10.1093/ejechocard/jep007 19270053

[21] Huttin O , Fraser AG , Coiro S , et al. Impact of changes in consensus diagnostic recommendations on the echocardiographic prevalence of diastolic dysfunction. J Am Coll Cardiol. 2017;69(25):3119‐3121. 28641802 10.1016/j.jacc.2017.04.039

[22] Ferreira JP , Girerd N , Bozec E , et al. Intima‐media thickness is linearly and continuously associated with systolic blood pressure in a population‐based cohort (STANISLAS cohort study). J Am Heart Assoc. 2016;5(6):e003529. doi:10.1161/JAHA.116.003529. 27312804 PMC4937282

[23] Van Bortel LM , Laurent S , Boutouyrie P , et al. Expert consensus document on the measurement of aortic stiffness in daily practice using carotid‐femoral pulse wave velocity. J Hypertens. 2012;30(3):445‐448. 22278144 10.1097/HJH.0b013e32834fa8b0

[24] Perrone‐Filardi P , Coca A , Galderisi M , et al. Noninvasive cardiovascular imaging for evaluating subclinical target organ damage in hypertensive patients: a consensus article from the European Association of Cardiovascular Imaging, the European Society of Cardiology Council on hypertension and the European Society of Hypertension. J Hypertens. 2017;35(9):1727‐1741. 28767484 10.1097/HJH.0000000000001396

[25] Aboyans V , Criqui MH , Abraham P , et al. Measurement and interpretation of the ankle‐brachial index: a scientific statement from the American Heart Association. Circulation. 2012;126(24):2890‐2909. 23159553 10.1161/CIR.0b013e318276fbcb

[26] Harrell F . Regression Modeling Strategies: with Applications to Linear Models, Logistic Regression, and Survival Analysis. New York, NY: Springer; 2001.

[27] Kannel WB . Elevated systolic blood pressure as a cardiovascular risk factor. Am J Cardiol. 2000;85(2):251‐255. 10955386 10.1016/s0002-9149(99)00635-9

[28] Li Y , Wei FF , Thijs L , et al. Ambulatory hypertension subtypes and 24‐hour systolic and diastolic blood pressure as distinct outcome predictors in 8341 untreated people recruited from 12 populations. Circulation. 2014;130(6):466‐474. 24906822 10.1161/CIRCULATIONAHA.113.004876 PMC4414316

[29] Devereux RB , Alderman MH . Role of preclinical cardiovascular disease in the evolution from risk factor exposure to development of morbid events. Circulation. 1993;88(4):1444‐1455. 8403291 10.1161/01.cir.88.4.1444

[30] Cui H , Wang F , Fan L , et al. Association factors of target organ damage: analysis of 17,682 elderly hypertensive patients in China. Chin Med J (Engl). 2011;124(22):3676‐3681. 22340223

[31] Lin JM , Hsu KL , Chiang FT , Tseng CD , Tseng YZ . Influence of isolated diastolic hypertension identified by ambulatory blood pressure on target organ damage. Int J Cardiol. 1995;48(3):311‐316. 7782147 10.1016/0167-5273(94)02239-f

[32] Bliziotis IA , Destounis A , Stergiou GS . Home versus ambulatory and office blood pressure in predicting target organ damage in hypertension: a systematic review and meta‐analysis. J Hypertens. 2012;30(7):1289‐1299. 22499289 10.1097/HJH.0b013e3283531eaf

[33] Korhonen PE , Syvanen KT , Vesalainen RK , et al. Ankle‐brachial index is lower in hypertensive than in normotensive individuals in a cardiovascular risk population. J Hypertens. 2009;27(10):2036‐2043. 19587608 10.1097/HJH.0b013e32832f4f54

[34] Powell TM , Glynn RJ , Buring JE , Creager MA , Ridker PM , Pradhan AD . The relative importance of systolic versus diastolic blood pressure control and incident symptomatic peripheral artery disease in women. Vasc Med. 2011;16(4):239‐246. 21730007 10.1177/1358863X11413166 PMC3154540

[35] Devereux RB , Pickering TG . Relationship between ambulatory and exercise blood pressure and cardiac structure. Am Heart J. 1988;116(4):1124‐1133. 2972182 10.1016/0002-8703(88)90176-7

[36] Missault LH , De Buyzere ML , De Bacquer DD , Duprez DD , Clement DL . Relationship between left ventricular mass and blood pressure in treated hypertension. J Hum Hypertens. 2002;16(1):61‐66. 11840231 10.1038/sj.jhh.1001295

[37] Fagard R , Staessen JA , Thijs L . The relationships between left ventricular mass and daytime and night‐time blood pressures: a meta‐analysis of comparative studies. J Hypertens. 1995;13(8):823‐829. 8557959 10.1097/00004872-199508000-00002

[38] Krittayaphong R , Boonyasirinant T , Saiviroonporn P , Thanapiboonpol P , Nakyen S , Udompunturak S . Correlation between NT‐pro BNP levels and left ventricular wall stress, sphericity index and extent of myocardial damage: a magnetic resonance imaging study. J Card Fail. 2008;14(8):687‐694. 18926441 10.1016/j.cardfail.2008.05.002

[39] Paget V , Legedz L , Gaudebout N , et al. N‐terminal pro‐brain natriuretic peptide: a powerful predictor of mortality in hypertension. Hypertension. 2011;57(4):702‐709. 21383312 10.1161/HYPERTENSIONAHA.110.163550

[40] Laurent S , Cockcroft J , Van Bortel L , et al. Expert consensus document on arterial stiffness: methodological issues and clinical applications. Eur Heart J. 2006;27(21):2588‐2605. 17000623 10.1093/eurheartj/ehl254

[41] Ngim CA , Abdul Rahman AR , Ibrahim A . Pulse wave velocity as an index of arterial stiffness: a comparison between newly diagnosed (untreated) hypertensive and normotensive middle‐aged Malay men and its relationship with fasting insulin. Acta Cardiol. 1999;54(5):277‐282. 10596307

[42] Di Bello V , Talini E , Dell'Omo G , et al. Early left ventricular mechanics abnormalities in prehypertension: a two‐dimensional strain echocardiography study. Am J Hypertens. 2010;23(4):405‐412. 20044741 10.1038/ajh.2009.258

[43] Galderisi M , Trimarco B . Speckle‐tracking echocardiography‐derived longitudinal dysfunction: a novel starting point of the hypertensive patient's journey toward heart failure. J Am Coll Cardiol. 2015;66(21):2472. 26610882 10.1016/j.jacc.2015.07.087

[44] Huttin O , Girerd N , Coiro S , et al. Association between layer‐specific longitudinal strain and risk factors of heart failure and dyspnea: a population‐based study. J Am Soc Echocardiogr. 2019;32(7):854‐865. 31104890 10.1016/j.echo.2019.03.011

