# Supplementary figures and images for: Isolated diastolic hypertension and target organ damage: Findings from the STANISLAS cohort
Source: Clin Cardiol. 2021 Sep 15;44(11):1516–25. doi: 10.1002/clc.23713 (PMC8571544; doi:10.1002/clc.23713)

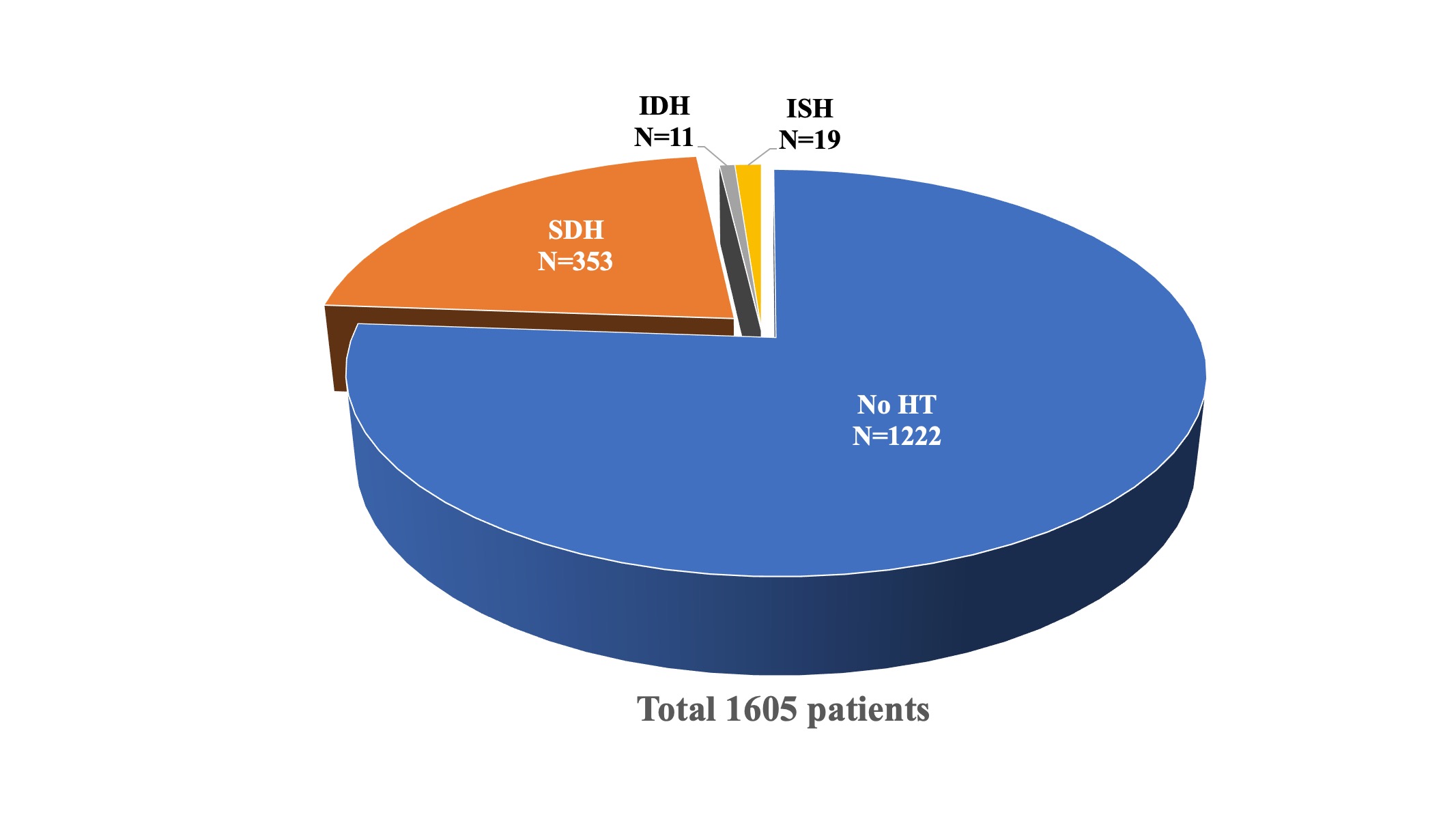

Supplement: Supplementary file 1 — Figure S1: Patients allocation according to hypertension categories defined by ESC/ESH criteria. Blue, no hypertension (no HT); orange, systolic‐diastolic hypertension (SDH); gray, isolated diastolic hypertension (IDH); yellow, isolated systolic hypertension (ISH). [file CLC-44-1516-s002.jpg]
