# Supplementary material for: Isolated diastolic hypertension and target organ damage: Findings from the STANISLAS cohort
Source: Clin Cardiol. 2021 Sep 15;44(11):1516–25. doi: 10.1002/clc.23713 (PMC8571544; doi:10.1002/clc.23713)
Supplement: Supplementary file 2 — Table S1: Crude and adjusted association between markers of target organ damage and hypertension categories (office BP measurement). [file CLC-44-1516-s001.doc]

**SUPPLEMENTAL MATERIAL**

**Supplemental Table 1.** Crude and adjusted association between markers of target organ damage and hypertension categories (office BP measurement).

|  |  | **Univariate** | | **Multivariable ^‡^** | |
| --- | --- | --- | --- | --- | --- |
| **Variable** | **Blood pressure categories** | **Odds ratio [95% CI]** | **p-value** | **Odds ratio [95% CI]** | **p-value** |
|  | *Reference: no hypertension* | 1.00 |  | 1.00 |  |
| **Left ventricular mass index, per increment of 10 g/m^2^** | Systolic-diastolic hypertension | 1.546 [1.446; 1.654] | <.0001^*^ | 1.194 [1.086; 1.313] | 0.0002^*^ |
|  | Isolated diastolic hypertension | 0.966 [0.785; 1.188] | 0.74 | 0.806 [0.640; 1.014] | 0.066 |
|  | Isolated systolic hypertension | 1.405 [1.293; 1.527] | <.0001^*^ | 1.185 [1.077; 1.304] | 0.0005^*^ |
| **Left ventricular hypertrophy, n (%)** | Systolic-diastolic hypertension | 3.039 [2.245; 4.115] | <.0001^*^ | 1.600 [1.040; 2.463] | 0.033^*^ |
|  | Isolated diastolic hypertension | 0.264 [0.036; 1.950] | 0.19 | 0.195 [0.026; 1.456] | 0.11 |
|  | Isolated systolic hypertension | 2.095 [1.388; 3.162] | 0.0004^*^ | 1.569 [0.999; 2.464] | 0.051 |
| **Left atrial volume index, per increment of 10 mL/m^2^** | Systolic-diastolic hypertension | 1.644 [1.411; 1.917] | <.0001^*^ | 1.366 [1.087; 1.716] | 0.007^*^ |
|  | Isolated diastolic hypertension | 0.523 [0.304; 0.900] | 0.019^*^ | 0.456 [0.257; 0.807] | 0.007^*^ |
|  | Isolated systolic hypertension | 1.362 [1.109; 1.674] | 0.003^*^ | 1.222 [0.968; 1.542] | 0.092 |
| **^†^ LV-GLS, %** | Systolic-diastolic hypertension | 1.858 [1.470; 2.347] | <.0001^*^ | 1.520 [1.078; 2.144] | 0.017^*^ |
|  | Isolated diastolic hypertension | 2.122 [1.105; 4.072] | 0.024^*^ | 1.881 [0.953; 3.710] | 0.068 |
|  | Isolated systolic hypertension | 1.362 [1.000; 1.854] | 0.05 | 1.021 [0.727; 1.433] | 0.91 |
| **^†^ Subendocardial LV-GLS, %** | Systolic-diastolic hypertension | 1.572 [1.247; 1.982] | 0.0001^*^ | 1.341 [0.952; 1.889] | 0.093 |
|  | Isolated diastolic hypertension | 1.719 [0.890; 3.318] | 0.11 | 1.563 [0.788; 3.100] | 0.2 |
|  | Isolated systolic hypertension | 1.215 [0.898; 1.644] | 0.21 | 0.947 [0.678; 1.323] | 0.75 |
| **E/e’ ratio** | Systolic-diastolic hypertension | 1.565 [1.458; 1.681] | <.0001^*^ | 1.144 [1.034; 1.264] | 0.009^*^ |
|  | Isolated diastolic hypertension | 1.049 [0.847; 1.301] | 0.66 | 0.946 [0.751; 1.192] | 0.64 |
|  | Isolated systolic hypertension | 1.245 [1.136; 1.365] | <.0001^*^ | 1.129 [1.017; 1.253] | 0.023^*^ |
| **E/A ratio** | Systolic-diastolic hypertension | 0.073 [0.050; 0.107] | <.0001^*^ | 0.397 [0.225; 0.700] | 0.001^*^ |
|  | Isolated diastolic hypertension | 0.243 [0.095; 0.622] | 0.003^*^ | 0.316 [0.099; 1.010] | 0.052 |
|  | Isolated systolic hypertension | 0.304 [0.202; 0.456] | <.0001^*^ | 0.662 [0.399; 1.099] | 0.11 |
| **Diastolic dysfunction, n (%)** | Systolic-diastolic hypertension | 5.146 [3.863; 6.855] | <.0001^*^ | 1.812 [1.185; 2.772] | 0.006^*^ |
|  | Isolated diastolic hypertension | 1.386 [0.528; 3.636] | 0.51 | 0.833 [0.297; 2.338] | 0.73 |
|  | Isolated systolic hypertension | 1.742 [1.136; 2.671] | 0.011^*^ | 1.026 [0.628; 1.677] | 0.92 |
| **LVEDV index, per increment of 10 mL/m^2^** | Systolic-diastolic hypertension | 1.007 [0.925; 1.097] | 0.86 | 1.023 [0.898; 1.165] | 0.74 |
|  | Isolated diastolic hypertension | 0.953 [0.738; 1.231] | 0.71 | 0.931 [0.704; 1.230] | 0.61 |
|  | Isolated systolic hypertension | 1.020 [0.910; 1.143] | 0.73 | 0.995 [0.872; 1.135] | 0.94 |
| **LVESV index, per increment of 10 mL/m^2^** | Systolic-diastolic hypertension | 0.909 [0.763; 1.082] | 0.28 | 1.121 [0.875; 1.436] | 0.37 |
|  | Isolated diastolic hypertension | 0.696 [0.392; 1.235] | 0.22 | 0.645 [0.343; 1.212] | 0.17 |
|  | Isolated systolic hypertension | 0.979 [0.779; 1.231] | 0.85 | 0.954 [0.731; 1.245] | 0.73 |
| **^†^ LV ejection fraction, %** | Systolic-diastolic hypertension | 1.640 [1.315; 2.046] | <.0001^*^ | 1.088 [0.784; 1.511] | 0.61 |
|  | Isolated diastolic hypertension | 1.446 [0.758; 2.759] | 0.26 | 1.350 [0.692; 2.632] | 0.38 |
|  | Isolated systolic hypertension | 1.493 [1.111; 2.008] | 0.008^*^ | 1.371 [0.991; 1.896] | 0.056 |
| **^†^Carotid intima media thickness, µm** | Systolic-diastolic hypertension | 4.363 [3.437; 5.537] | <.0001^*^ | 1.052 [0.723; 1.531] | 0.79 |
|  | Isolated diastolic hypertension | 2.303 [1.206; 4.395] | 0.011^*^ | 1.487 [0.671; 3.298] | 0.33 |
|  | Isolated systolic hypertension | 2.740 [2.017; 3.721] | <.0001^*^ | 1.715 [1.166; 2.524] | 0.006^*^ |
| **Pulse wave velocity, m/s** | Systolic-diastolic hypertension | 1.956 [1.793; 2.133] | <.0001^*^ | 1.509 [1.336; 1.703] | <.0001^*^ |
|  | Isolated diastolic hypertension | 1.368 [1.092; 1.715] | 0.006^*^ | 1.138 [0.875; 1.479] | 0.34 |
|  | Isolated systolic hypertension | 1.716 [1.552; 1.898] | <.0001 | 1.435 [1.268; 1.623] | <.0001 |
| **Microalbuminuria, per increment of 100 mg/L** | Systolic-diastolic hypertension | 1.329 [0.981; 1.802] | 0.067 | 0.989 [0.717; 1.365] | 0.95 |
|  | Isolated diastolic hypertension | 1.082 [0.418; 2.803] | 0.87 | 1.003 [0.557; 1.806] | 0.99 |
|  | Isolated systolic hypertension | 0.805 [0.372; 1.744] | 0.58 | 0.875 [0.513; 1.492] | 0.62 |
| **^†^ C-reactive protein, per increment of 10 mg/L** | Systolic-diastolic hypertension | 1.892 [1.521; 2.354] | <.0001 | 1.274 [0.910; 1.783] | 0.16 |
|  | Isolated diastolic hypertension | 1.271 [0.674; 2.397] | 0.46 | 1.201 [0.606; 2.378] | 0.6 |
|  | Isolated systolic hypertension | 1.355 [1.010; 1.818] | 0.043 | 1.252 [0.900; 1.742] | 0.18 |
| **^†^ NT-ProBNP, (Olink)** | Systolic-diastolic hypertension | 1.432 [1.151; 1.781] | 0.001 | 1.184 [0.834; 1.679] | 0.35 |
|  | Isolated diastolic hypertension | 0.589 [0.298; 1.166] | 0.13 | 0.464 [0.214; 1.003] | 0.051 |
|  | Isolated systolic hypertension | 1.044 [0.778; 1.402] | 0.77 | 1.149 [0.808; 1.634] | 0.44 |

Abbreviations: BP, blood pressure; GLS, global longitudinal strain; LV, left ventricular; LVEDV, left ventricular end-diastolic volume; LVESV, left ventricular end-systolic volume.

^†^ log linearity assumption not respected. Variable categorized according to the median value (≥ median).

^‡^ Multivariate adjusted for: sex, age, waist circumference, smoking status, total cholesterol, glycemia, lipid lowering agents, hypertension status, glomerular filtration rate and hemoglobin.

^*^ Statistically significant.
